# Practical Applications of 2D Material FET Biosensors: Functionalization Strategies and Detection Performance

**DOI:** 10.3390/bios16060304

**Published:** 2026-05-23

**Authors:** Binbin Gao, Guohui Li, Milica Balaban, Vesna Antic, Muhammad Zeeshan Tahir, Li Gao

**Affiliations:** 1 School of Life Sciences, Jiangsu University, Zhenjiang 212013, China; 2 Faculty of Natural Sciences and Mathematics, University of Banja Luka, Mladena Stojanovića 2, 78000 Banja Luka, Bosnia and Herzegovina; 3 Faculty of Agriculture, University of Belgrade, Nemanjina 6, 11080 Belgrade, Serbia; 4 School of Materials Science and Engineering, Jiangsu University, Zhenjiang 212013, China

**Keywords:** 2D material FET biosensors, biosensing, surface functionalization, real-sample detection, practical applications

## Abstract

Two-dimensional-material-based FET biosensors have gained attention for being label-free and having ultra-sensitive detection capability. The high carrier mobility and large surface-to-volume ratio of 2D materials enable low detection limits under buffer conditions; however, practical detection still faces many challenges. Current reviews have largely summarized materials, functionalization routes, or target classes separately, but a clearer framework linking interface design, device architecture, and practical sensing performance is still needed. In this review, we examine how interfacial engineering and device architecture govern signal transduction and sensing behavior in 2D material FET biosensors. We also analyze the major barriers to real-sample detection, including Debye screening, nonspecific adsorption, and signal drift, together with commonly used mitigation strategies. On this basis, an “interface–device–performance” framework is discussed as a conceptual approach for understanding the relationship between molecular recognition, electrical response, and sensing performance. This review mainly focuses on the key challenges of 2D material FET biosensors in practical medical applications, discusses the differences between material and application perspectives, and examines the major factors limiting clinical translation.

## 1. Introduction

With the growing emphasis on early diagnosis, personalized therapy, and continuous health monitoring, the demand for precision medicine and real-time analytical detection technologies continues to rise accordingly [1,2,3]. In this context, sensitive and rapid biomolecular detection has become increasingly important for clinical diagnosis, disease monitoring, and health management. In complex detection environments, detecting ultra-low concentrations of biomolecules is currently a key challenge that biosensors need to address [4,5]. Field-effect transistor (FET) biosensors offer several advantages, such as label-free detection, rapid response, and ease of integration [6,7]. These features make FETs an attractive platform for biosensing. Integrating two-dimensional materials with high surface area and high carrier mobility, such as graphene and molybdenum disulfide, into this platform can significantly enhance sensor sensitivity and response performance [8,9,10]. Two-dimensional materials are atomically thin materials with highly exposed surfaces and ultrathin conductive channels. Compared with conventional bulk materials, their electrical properties are more sensitive to interfacial charge changes, making them particularly suitable for electrical biosensing applications.

Despite rapid progress in two-dimensional-material FET biosensors, several challenges remain [11]. A substantial body of research has focused on surface functionalization and reported high sensitivity under controlled conditions, but systematic evaluation in real samples remains limited [8,12,13,14]. As a result, high analytical performance reported in buffer systems is often difficult to reproduce in complex biological matrices. At a fundamental level, the performance of two-dimensional-material FET biosensors is governed by two closely related aspects: interfacial functionalization and device-level physical regulation. Functionalization defines how biorecognition molecules are immobilized at the sensing interface and therefore strongly influences sensor selectivity [15]. When target molecules selectively bind to the recognition layer, they can change interfacial electrical properties such as charge distribution and local dipole configuration [16]. These interfacial changes are then converted into measurable electrical responses by the device. However, interfacial biological processes remain a major source of difficulty in practical detection. Under a high-ionic-strength environment, Debye screening can weaken the electrostatic signal transduction, while nonspecific adsorption can cause signal drift and even false-positive or false-negative results [17,18,19]. Both interface regulation and device-level engineering need to be considered together when evaluating practical sensing performance.

In recent years, novel device architecture strategies such as heterostructure engineering, light-assisted signal amplification, and dual-gate tuning have further enhanced the sensitivity of FET biosensors to subtle electrical perturbations [20,21,22]. However, these methods are still seldom integrated systematically with established functionalization strategies. This separation has limited a more unified understanding of how interfacial events are converted into measurable device responses.

**Figure 1:**
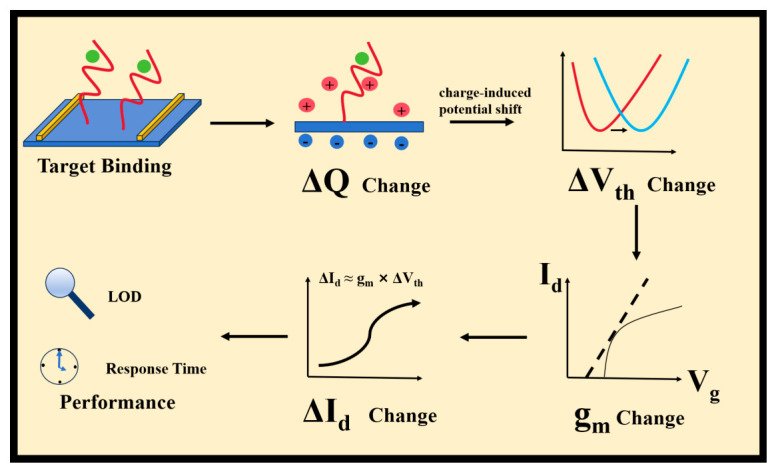
Schematic illustration of the interface–device–performance coupling framework in 2D-material FET biosensors.

This review focuses on how interfacial engineering and device architecture jointly influence signal transduction and sensing performance in 2D-material FET biosensors, with particular emphasis on how interfacial processes shape device response and detection performance [9]. Unlike reviews that focus on materials and applications, this article provides an application approach based on a materials science, with a particular emphasis on how interface changes affect the electrical behavior of devices and further impact sensing performance, ultimately serving practical detection and providing a unified understanding for interdisciplinary readers. At the same time, this article attempts to point out and correct the differences in understanding caused by different perspectives: for example, in biological analysis, diffusion and reaction processes are involved, while in material perspectives, they are often simplified as direct electrical responses, resulting in cognitive biases. Based on the research progress of 2D-material FETs in nucleic acid, protein, and small-molecule detection in recent years, this review summarizes and analyzes typical interface construction methods and device structure design, and discusses their roles in signal transduction and practical detection [23,24,25]. On this basis, this article proposes an “interface–device–performance” framework to interpret the relationship between molecular recognition events and electrical signal output (Figure 1), and explores the applicability of different strategies in complex environments [11,26]. This framework is mainly intended to help connect interfacial processes, device electrical behavior, and sensing performance for readers from different research backgrounds. Due to the complexity of liquid-gated systems and interfacial environments, it should be understood as a conceptual or semi-quantitative description rather than a universal predictive model.

## 2. Functionalization Strategies and Deposition Methods of 2D Materials for FET Biosensors

### 2.1. Deposition Methods of 2D Materials onto Substrates

The deposition method of 2D materials onto substrates can strongly affect the electrical properties, interface quality, and sensing stability of FET biosensors [9,19]. Although many current studies mainly focus on surface functionalization and biomolecular recognition strategies, the fabrication process of the semiconductor channel itself is also an important factor influencing device reproducibility and long-term performance [26]. At present, chemical vapor deposition (CVD) is one of the most commonly used preparation methods for 2D materials because it enables controllable large-area growth. As a result, it has been widely used in graphene and MoS_2_ based FET devices [9]. However, CVD-grown 2D materials usually need to be transferred from metal growth substrates onto Si/SiO_2_, glass, or flexible substrates. During this process, PMMA is commonly used as a supporting layer. PMMA residues, wrinkles, cracks, and trapped impurities can introduce additional charge-scattering centers, which may affect carrier transport behavior and device stability. Previous studies have shown that PMMA residues can cause noticeable Dirac point shifts and reduce carrier mobility. To reduce surface contamination, researchers have explored different cleaning methods, including acetone, acetic acid, thermal annealing, electron-beam treatment, ultraviolet ozone treatment, and plasma cleaning. Under certain experimental conditions, electron beam-assisted cleaning showed better PMMA removal efficiency and smaller Dirac point shifts after cleaning [27]. However, this conclusion may not always apply to other systems because device structure, substrate type, dielectric environment, and fabrication conditions can all influence the final electrical behavior. Mechanical exfoliation is difficult to use for large-area fabrication, but it usually produces 2D materials with fewer defects and relatively high carrier mobility. Since most 2D FET biosensors do not require very large active areas, mechanically exfoliated materials are still widely used in many studies, especially in device mechanism research. In comparison, liquid-phase exfoliation and solution-based fabrication methods offer advantages such as lower cost and compatibility with flexible and printable electronics. However, the obtained materials often suffer from nonuniform layer thickness, relatively high defect density, and poorer electrical uniformity. Direct growth on insulating substrates may reduce transfer-related contamination and improve interfacial uniformity, which can help improve long-term device stability and reduce device-to-device variation. Nevertheless, these methods usually require relatively high growth temperatures and may still face compatibility issues with flexible substrates or certain biological systems [26].

At present, many studies still mainly emphasize ultralow limits of detection (LODs) and short-term sensing responses, while discussions regarding long-term electrical drift, device variation, and fabrication consistency caused by deposition processes remain relatively limited. Future studies should further correlate deposition methods, interfacial properties, and device-level electrical parameters to improve the practical applicability of 2D-material FET biosensors.

### 2.2. Covalent Functionalization

**Figure 2:**
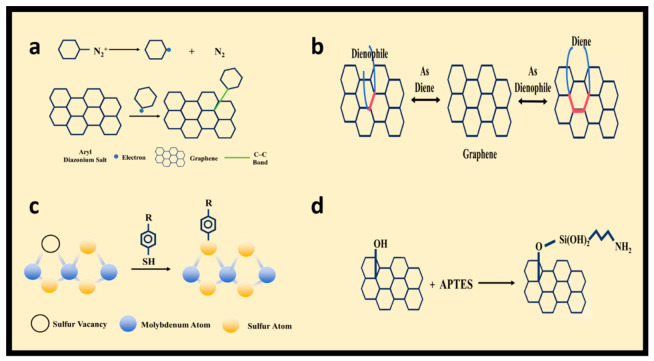
Representative covalent functionalization routes and their interfacial bonding mechanisms in 2D materials. (**a**) Diazonium-induced radical grafting on graphene, introducing sp^3^ defects. Adapted from ref. [29]. (**b**) Diels–Alder cycloaddition on graphene basal plane. Adapted from Ref. [37]. (**c**) Thiol-mediated defect functionalization at MoS_2_ sulfur vacancies. Adapted from Ref. [47]. (**d**) APTES silanization on graphene oxide via Si–O–C linkage. Adapted from Ref. [49].

Radical chemistry represents a commonly employed covalent modification strategy for two-dimensional materials. Radicals represented by aromatic diazonium salts can release active aryl radicals, which can form stable C–C covalent bonds with the sp^2^ carbon network [28,29,30]. This method exhibits high chemical stability. From an electronic perspective, radical grafting introduces sp^3^ defects into the graphene lattice, disrupts the π-electron conjugation, increases carrier scattering, and reduces mobility [30,31,32,33]. The reaction mechanism of covalent functionalization between aryl diazonium salt and graphene via radical pathway is illustrated in Figure 2a.

Cycloaddition reactions, including Diels–Alder and [3+2] cycloaddition reactions, usually preserve more of the original conjugated structure compared with radical-based functionalization methods [34,35]. These reactions typically occur at specific reaction sites and are capable of introducing functional groups under relatively mild conditions. From an electronic perspective, cycloaddition reactions induce local band-structure modulation without extensively converting sp^2^ carbon atoms to sp^3^ [35,36]. As a result, the reduction in carrier mobility is often smaller than that induced by radical functionalization. In Diels–Alder [4+2] cycloaddition, graphene can act as either a dienophile, reacting with electron-rich dienes in the Diels–Alder [4+2] cycloaddition reaction, or as a diene, undergoing concerted addition with electron-deficient dienophiles. In Figure 2b, the highlighted red atoms represent carbon atoms in the graphene basal plane that participate in cycloaddition. These atoms contribute π electrons to form two new C-C σ bonds with the reaction partner, converting the local hybridization from sp^2^ to sp^3^ while preserving most of the π-conjugated network [37,38].

Michael addition and nucleophilic addition reactions are commonly used to functionalize defect sites or oxidized regions of graphene oxide (GO) and transition metal disulfides [39,40,41]. Such reactions mainly target oxygen-containing groups or sulfur vacancies on the two-dimensional material surface and therefore do not cause extensive material surface modification. In the MoS_2_ systems, functionalization at sulfur vacancies has been reported to influence contact resistance and may affect the carrier transfer behavior, which can in turn influence the threshold-voltage behavior [42,43,44,45]. Organic molecules containing thiol groups (R-SH) can react at sulfur-vacancy sites to form stable Mo–S–R covalent bonds with molybdenum atoms, thereby enabling local functionalization. The yellow and blue spheres in Figure 2c represent sulfur atoms and molybdenum atoms, respectively. Through this defect-mediated covalent bonding, the 2D material surface can be chemically modified in a localized manner without extensive disruption of the lattice structure [46,47].

Silanization is a commonly employed covalent functionalization strategy. For instance, APTES can form a self-assembled monolayer (SAM) on oxide surfaces, introducing terminal functional groups and interfacial dipole layers at the dielectric/semiconductor interface [48]. The GO surface contains abundant oxygen-containing groups, which can condense with silanol species generated by hydrolysis of silane coupling agent to form stable Si–O–C covalent bonds, thereby enabling covalent grafting of silane groups onto the GO surface [49,50,51]. Figure 2d schematically illustrates the reaction between hydroxyl (-OH) groups of APTES and oxygen-containing groups on graphene oxide. After APTES functionalization of the GO surface, terminal NH_2_ groups are retained, which helps subsequent immobilization of the biorecognition molecules to achieve target detection.

As a common covalent functionalization method, polymer grafting can improve biocompatibility and antifouling performance. Polyethylene glycol (PEG) and poly-L-lysine (PLL) are commonly used examples [52]. The polymer layer can increase the effective sensing distance between the analyte and the conductive channel. Although this can reduce nonspecific adsorption and improve stability, it can also weaken electrostatic coupling under Debye-screening conditions [53]. In addition, polymer functionalization can affect interfacial capacitance and alter gate efficiency in liquid-gated systems [54]. PEG can react with carboxyl (-COOH) groups on the surface of graphene oxide to form covalent bonds, after which the resulting PEG-functionalized GO can be further coupled to other biorecognition molecules.

In addition, covalent functionalization can also be achieved by utilizing oxygen-containing groups on the surface of graphene oxide, such as -COOH, which allows for the subsequent amide-bond formation with amine-modified biomolecules [55,56]. Overall, covalent functionalization provides a chemically robust method for integrating recognition chemistry with two-dimensional materials. Although this approach offers high stability, it can also disrupt the lattice structure of 2D materials and increase carrier scattering, which may further reduce carrier-transport performance [57].

### 2.3. Noncovalent Functionalization

Noncovalent functionalization preserves the intrinsic lattice structure and carrier mobility of two-dimensional materials. Instead of forming covalent bonds, noncovalent functionalization relies on weak intermolecular interactions [58,59].

π–π stacking is widely used in two-dimensional-material FET biosensors, especially in graphene-based systems. A typical example is pyrenebutanoic acid succinimidyl ester (PBASE), which can be adsorbed on the graphene surface through π–π stacking without damaging the sp^2^ lattice [60]. From an electronic perspective, π–π stacking introduces minimal structural disruption, thereby preserving carrier mobility and limiting additional scattering. However, surface-charge redistribution and dipole formation introduced by linker molecules can modify the local electrostatic potential, resulting in measurable threshold-voltage shifts [60,61]. Under this simplified description, ΔVth can be regarded as an indicator of interfacial charge perturbation and its influence on electrical response. For instance, previous studies have reported that PBASE functionalization alone can induce a Dirac point shift of approximately 75 mV, indicating that this functionalization method can induce effective interface charge transfer and Fermi-level modulation [62].

Electrostatic adsorption utilizes Coulomb interactions between charged biomolecules and oppositely charged 2D surfaces [8]. This method can enable rapid and reversible probe immobilization under mild conditions. However, due to its mild interaction, it is susceptible to nonspecific adsorption and changes in ion environments in complex systems, thus limiting its stability and selectivity in actual samples. Electronically, electrostatic adsorption mainly changes the surface charge density. The sensing mechanism usually relies on the direct modulation of the channel charge density, resulting in an observable threshold-voltage shift [11,35]. This behavior can also be explained within the proposed framework as a direct coupling between interface charge changes and electrical output.

Functionalization is a key step in interface engineering, and covalent and noncovalent strategies are two main approaches. Although they may cause changes in threshold voltage (ΔVth), their more important role is to achieve stable fixation of target molecules. Covalent methods provide strong chemical stability, making them especially suitable for the detection of complex biological fluids or long-term operation. However, these methods usually introduce lattice perturbation or dielectric modification, which reduces carrier mobility and increases scattering, thus affecting device performance [63,64,65,66]. The noncovalent strategy preserves the intrinsic electronic structure of the 2D channel and maintains high carrier mobility. These methods are beneficial to achieve high intrinsic sensitivity and low noise, especially in low-ionic-strength environments. Their limitations become more apparent in physiological media, where weaker interfacial stability and stronger Debye screening can reduce practical applicability. Table 1 summarizes the comparative features of these functionalization strategies in 2D-material FET biosensors. During functionalization, biorecognition molecules can be positioned farther from the channel by using longer linker molecules [22,67,68]. If the recognition layer becomes too thick and the distance between the target charge and the channel exceeds the Debye length in solution, the electric-field modulation effect is significantly weakened [69,70,71]. Accordingly, Table 1 systematically compares mobility retention, threshold-voltage modulation, Debye-screening tolerance, and operational stability, providing a device-oriented basis for rational sensor design.

In most biosensing studies, the electrical contribution of functionalization is not examined in isolation; rather, the effect of functionalization is usually inferred from changes in transfer characteristics, including Dirac-point shift, current variation, and signal stability. A simple way to describe the voltage shift induced by functionalization is as follows:

ΔVth = ΔQ/Ceff (for graphene-based and related systems, VDirac may be used in place of ΔVth).

Here, ΔQ denotes the interfacial charge change, and Ceff is the effective capacitance. Under this simplified description, stronger charge transfer or dipole effects lead to larger electrical responses [72]. However, it should be noted that the relationship above is mainly intended to conceptually describe the connection between interfacial charge variation and device response, rather than serve as a rigorous quantitative predictive model. In practical liquid-gated FET systems, Ceff can be dynamically influenced by factors such as electrolyte concentration, gate bias, interfacial dipole configuration, and ionic environment, and therefore cannot be regarded as strictly constant.

In practical sensing studies, several factors usually need to be considered during functionalization. The induced voltage shift should be large enough to be measured, but not so excessively large that the device stability is compromised. Carrier transport should also be largely preserved, which may be evaluated by ratio μ/μ0. Here, μ0 and μ denote the mobility before and after functionalization, respectively. In addition, Debye-length constraints may provide a useful qualitative guideline:

d/λD ≤ 1

This is particularly important because Debye screening is still one of the main factors limiting sensor performance, and it is necessary to minimize the effective operating distance while ensuring interface stability.

Taken together, functionalization is better determined by considering voltage modulation, transport preservation, and interfacial stability together rather than relying on any single parameter [72]. Among these factors, the ability to achieve stable probe fixation and reliable target binding is particularly crucial, as it fundamentally determines the effectiveness of subsequent signal transduction.

## 3. Practical Applications of 2D-Material FET Biosensors

After discussing the functional strategies and their electronic modulation effects, the next question is how these design principles translate into practical sensing performance. 2D-material FET biosensors have been applied to a wide range of targets, including nucleic acids, proteins, and small-molecule or ion analytes, and have demonstrated extremely high detection performance in laboratory environments, including in buffer solutions [14,58]. Reported detection limits for nucleic acids and proteins can reach as low as femtomolar range or below, reflecting the strong signal-transduction capability of FET-based platforms [20]. This section therefore organizes representative applications by target type and compares key performance metrics, including detection limit, linear range, and response behavior. Where available, performance in real samples is also noted to distinguish proof-of-concept sensing from more application-oriented studies [70]. Most of these performance values were obtained under simplified experimental conditions and may not translate directly to real biological or environmental samples. This limitation is discussed further in the next section.

### 3.1. Nucleic Acids

Nucleic acids, particularly DNA and RNA, are major targets in biosensing because of their relevance to gene regulation and disease diagnosis. In 2D-material FET biosensors, nucleic-acid detection typically relies on sequence-specific hybridization between probes immobilized on the channel surface and complementary targets in solution. Hybridization redistributes interfacial charge and can therefore be read out electrically through changes in device characteristics [73,74]. Recent work has focused not only on lowering the detection limit, but also on improving signal retention in complex media through interface engineering and device design.

Hwang et al. reported a wrinkled graphene FET with detection limits down to approximately 600 zM in buffer and 20 aM in human serum samples [73]. The wrinkled graphene structure enhanced interfacial charge coupling and improved electrical response under high-ionic-strength environments. Detectable Dirac-point shifts were still observed in undiluted human serum; however, the sensing performance was reduced compared with buffer conditions, indicating that matrix effects and Debye screening still affect sensing performance in complex biological environments. At present, this study mainly demonstrated the feasibility of detection in real biological samples, while linear range, long-term stability, and quantitative sensing performance in complex environments still require further evaluation [73,75]. Wang et al. introduced a graphene/MoS_2_ heterojunction field-effect transistor (FET) biosensor for highly sensitive miRNA detection [76]. In this design, the recognition interface was constructed on a two-dimensional material surface to allow stable probe immobilization and sequence-specific electrical signal readout. Relative to traditional single-material devices, the heterojunction structures effectively improved interfacial charge coupling and carrier control, which contributed to better device stability and integration. In addition, this study was mainly performed under buffer conditions, while sensing performance in complex biological environments still requires further validation.

Hu et al. developed a biosensor based on liquid-gated GFET for rapid detection of miRNA-208a, and this biosensor exhibits femtomolar-level sensitivity [77]. This device achieves stable immobilization of probe molecules by constructing a nucleic-acid recognition interface on the graphene surface, thereby realizing sequence-specific recognition and electrical transduction of target miRNAs. In addition, this work, combined with a portable detection system, achieved miniaturization of the device and on-site detection capability. Xing et al. developed a field-effect transistor biosensor based on ReS_2_-MoS_2_ heterojunction nanotubes for ultrasensitive detection of miRNA-21 [78]. This device uses ReS_2_-MoS_2_ nanotubes with a superlattice structure as channel material and achieves efficient immobilization of probe molecules through a high-specific-surface-area structure, thereby enabling specific recognition of nucleic acid molecules and electrical-signal transduction. Compared with traditional two-dimensional material channels, this heterojunction structure can effectively suppress electron–hole recombination and enhance carrier transport capability. When combined with light-assisted regulation, it further improves signal response and achieves synergistic optimization of electrical and sensing performance. The device also demonstrated good stability, with recovery values ranging from 95.7% to 101.3% in complex biological environments. Considering that miRNA-21 concentrations in clinical samples are often already within the fM–pM range, while the detection limit in this work has reached 2.1 aM, further lowering the detection limit alone may have limited practical benefit. Future work may need to focus more on long-term stability, quantitative detection performance, and validation in real clinical samples.

Zhao et al. developed a transistor array biosensor based on electrochemically reduced graphene oxide (rGO) for the detection of multiple miRNAs associated with non-small-cell lung cancer [79]. The device uses rGO as the channel material and introduces a PAA/PEDOT composite functional layer on its surface to provide carboxyl groups for stable immobilization of capture DNA. A Y-shaped nucleic acid structure is designed to enhance the recognition efficiency and signal response of the target miRNA. Compared to traditional single-channel devices, this array structure achieves simultaneous multi-target detection and effectively improves selectivity and signal amplification capability through interface polymer modification, while also offering good reusability. This sensor exhibits excellent detection performance, with low detection limit, wide linear range, and good stability and selectivity, demonstrating potential for clinical liquid-biopsy applications. In human serum samples, it showed good stability with a recovery range of 91.1% to 108.8% and a relative standard deviation (RSD) of 2.2–4.1%.

Zhang et al. reported an MXene-based FET biosensor incorporating platinum nanowires for the detection of extracellular-vesicle miRNA-21 [80]. The platinum nanostructures significantly improved channel conductivity and transconductance, which in turn enhanced charge transfer and signal amplification. This metal/2D material composite achieved highly sensitive detection and also performed well in clinical samples, yielding a correlation coefficient of R2 = 0.8529 and an area under the curve (AUC) of 0.904. Compared with the nanophotonic biosensor reported by Calvo-Lozano et al., which showed a detection limit of approximately 25 pM for miRNA-21, 2D FET biosensors can reach much lower limits, down to the fM–aM range, demonstrating a significant improvement in detection performance. These studies indicate that two-dimensional FET biosensors have excellent sensitivity and rapid response capability in nucleic-acid detection, demonstrating their strong signal-transduction ability.

Taken together, the recent developments in 2D material-based FET biosensors for nucleic-acid detection have focused on interface engineering, device architecture, and signal amplification, significantly improving sensitivity and selectivity, while in some cases, they have also achieved portable formats and multi-channel detection in practical applications [81]. Notably, improvements in analytical sensitivity do not always imply equivalent performance in complex matrices, and this remains an important issue for practical translation.

### 3.2. Proteins

Proteins are important biomarkers in various physiological processes and in disease monitoring. Compared with nucleic acids, protein detection is usually more challenging due to their complex structure, larger size, and uneven surface-charge distribution [6,13]. However, 2D-material FET biosensors have shown strong potential in protein detection due to their high sensitivity, label-free operation, and real-time response [9]. In most cases, protein sensing relies on specific recognition elements, such as antibodies or aptamers immobilized on the channel surface, where the binding of target proteins induces changes in interfacial charge distribution, which can be directly converted into electrical signals [82]. Wang et al. developed an FET biosensor for cytokine detection (IFN-γ) using aptamer functionalized MoS_2_, in which lipoic acid served as a linker to immobilize the aptamer on the surface of MoS_2_ [83]. The introduction of defect sites through argon (Ar) etching increased the density of active binding sites, thereby improving probe immobilization and signal transduction efficiency, and enhancing sensing performance. The signal response of etched MoS_2_-FET biosensors is about 2.84-fold and 5.21-fold higher than that of unetched MoS_2_-FET and graphene–FET under the same detection conditions, respectively. For example, conventional ELISA-based detection of IFN-γ typically exhibits a detection limit of approximately 1 pg/mL, corresponding to the pM level, whereas 2D FET biosensors have achieved detection down to the fM level, representing an improvement of several orders of magnitude in sensitivity [84]. Wen et al. reported a MoS_2_-based FET biosensor for Tau protein detection by optimizing the biological functionalization strategy [85]. The study systematically compared different surface-modification methods to improve antibody immobilization efficiency and interfacial coupling, thereby enhancing sensitivity and stability, and achieved a detection limit of 0.01 pg/mL over a wide concentration range. Fathi-Hafshejani et al. developed a two-dimensional FET biosensor based on single-layer WSe_2_ for detecting the spike protein of severe acute respiratory syndrome coronavirus 2 [86]. In this system, the WSe_2_ channel was functionalized with specific antibodies through MUA linkers, enabling selective target recognition. During antigen binding, the device generated a clear electrical response, with a reported detection limit of 25 fg μL^−1^ and real-time signal readout.

Nisar et al. reported a MoTe_2_-FET biosensor for rapid detection of streptavidin [87]. In their design, engineered support structures were introduced to facilitate the immobilization of biomolecules. In their design, a pyrene lysine biotin (PLB) carrier structure was employed, in which pyrene was anchored on MoTe_2_ with a lysine linker providing spacing, and biotin served as a specific binding site for streptavidin protein. Due to interface charge transfer, this functionalization step introduces an initial transition in transfer characteristics, and subsequent streptavidin binding further alters the channel charge distribution, resulting in additional modulation of threshold behavior ΔVth and overall electrical response. The device achieved rapid detection within a response time of about 20 s and reached a detection limit of 1 pM. The sensor also exhibited good repeatability and stability over multiple measurement cycles, indicating its reliability and practical potential for biosensing applications. Chen et al. developed a vertical MoS_2_/graphene heterostructure FET biosensor for biomolecule detection [20]. Different biological functionalization strategies were applied on the two layers, including silane-based modification on MoS_2_ and π-π-interaction-assisted immobilization on graphene, allowing efficient probe attachment and interfacial engineering. This heterogeneous structure combines the high carrier mobility of graphene with the semiconducting properties of MoS_2_, which strengthens interfacial charge coupling and signal transduction. An important aspect of this work was the comparison between two stacking configurations, graphene-on-MoS_2_ (GM) and MoS_2_-on-graphene (MG), which showed that the GM configuration exhibited better biosensing performance. However, studies on protein and other large biomolecule detection using 2D-material FET biosensors are still much less common than those focused on small nucleic-acid targets such as DNA and miRNA. In many cases, protein sensing is still mainly carried out under buffer conditions, while sensing stability, reproducibility, and quantitative performance are often lower than those observed in small-molecule systems. This is largely related to the larger molecular size, more complex structures, and nonuniform surface-charge distribution of proteins. Under physiological conditions, these binding events often already occur beyond the Debye length, which weakens effective coupling between interfacial charges and the conductive channel. In addition, the interfacial electrical perturbations induced by large biomolecules are usually more complex and less stable, further increasing the difficulty of reliable electrical signal readout and signal discrimination. As a result, compared with nucleic-acid sensing, protein detection often relies more heavily on the combined optimization of interface engineering, local electric-field regulation, and device-level signal-amplification strategies.

### 3.3. Small Molecular and Ionic Targets

Small molecules and ions, such as hormones, neurotransmitters, and metal ions, play a crucial role in physiological regulation, whereas some metal ions can be harmful to the human body and are difficult to eliminate [6]. Because these analytes are often present at low concentrations, their accurate detection and requires high sensitivity and strong anti-interference capability [13,71]. Lee et al. developed a black phosphorus (BP)-based FET biosensor platform for cortisol detection in artificial saliva [88]. In this work, antibody-coupled magnetic particles were introduced to achieve reversible binding and release of target molecules, allowing reuse of the sensing interfaces. The BP channel provides high carrier mobility and strong surface sensitivity, enabling real-time and reusable detection of cortisol in biological samples.

Kim et al. developed a GFET biosensor for cortisol detection by immobilizing scFv-Fc antibodies through oligomeric (phenylethynyl)amine (OPE) interfacial linkers [89]. The covalent functionalization between OPE and graphene enhanced the carrier mobility and increased the binding affinity between the antibodies and cortisol, thereby achieving effective signal transduction. The device exhibited high selectivity and was able to detect cortisol in artificial sweat, with performance comparable to that under standard conditions, indicating potential for practical wearable-monitoring-device fabrication. Meng et al. developed a gold nanoparticle (AuNP)/3D crumpled graphene-based field-effect transistor biosensor for dopamine detection [90]. The synergistic effect of wrinkled graphene and gold nanoparticles enhanced charge transfer, reduced Debye screening, and enabled efficient signal transduction, together with ultrasensitive detection in complex biological environments. Detection limits of 60, 240, and 316 zM were reported in PBS, urine, and serum, respectively, indicating robust performance across complex biological matrices.

In addition to hormone and biomarker detection, 2D material-based FET biosensors have also been widely explored for heavy-metal-ion sensing. For example, a graphene FET array biosensor was developed for ultrasensitive Hg^2+^ detection using ssDNA probes. In the selectivity tests, even when competing ions such as Cu^2+^, Pb^2+^, Cr^3+^, and Cd^2+^ were present at concentrations two-orders-of-magnitude higher than Hg^2+^, the device still showed almost no obvious response until 100 pM Hg^2+^ was introduced, indicating good selectivity toward Hg^2+^. The sensing mechanism mainly relied on the specific interaction between Hg^2+^ and the DNA probes, which induced interfacial charge changes and further modulated the channel conductance through the field effect [91]. Recently, some emerging 2D-material-sensing platforms have started to adopt different design strategies. For example, Hg^2+^ sensing based on nonlayered ultrathin-β-In_2_S_3_ materials achieved ultrahigh sensitivity by constructing surfaces with abundant highly active sites. This system mainly relied on selective Hg–S interactions to enhance Hg^2+^ adsorption and charge transfer at the material surface. Compared with conventional FET biosensors that mainly depend on gate modulation and stable carrier transport for signal amplification, these systems place greater emphasis on direct interfacial interaction between the analyte and the sensing material. Although this platform is not a strict FET architecture, the idea of enhancing sensing signals through interfacial adsorption and charge transfer may still provide useful inspiration for future 2D-material FET biosensors [92]. For example, introducing controllable active sites, local defect regions, or engineered high-activity interfaces onto the FET channel surface may strengthen analyte–channel coupling and further improve sensing sensitivity. However, such strategies may also introduce more defect states, charge-trapping effects, and electrical drift. As a result, while sensitivity can be significantly improved, device selectivity and long-term stability may be partially sacrificed.

Taken together, the studies summarized in Table 2 show that 2D material-based FET biosensors can perform well across several target classes, including nucleic acids, proteins, and small molecules or ions [60]. For nucleic acids, detection is usually based on sequence-specific hybridization, where changes in interfacial charge can be converted into electrical signals. This structure is relatively simple and makes it easier to obtain lower detection limits [73]. Protein sensing is often more challenging because proteins are larger, structurally complex, and more heterogeneous in charge distribution, thus placing stricter requirements on interfacial compatibility, recognition specificity, and signal stability [53]. Contrary to proteins, small molecules and ions present a different challenge: they are often present at low concentrations and are more easily affected by complex sample matrices, so their reliable detection requires both high sensitivity and strong resistance to interference [71].

Although two-dimensional material FET biosensors are often described as being label-free and have rapid detection capabilities, in practical experiments, their detection time may be longer. This difference mainly stems from different understandings of “response time”. From the perspective of materials or devices, response time usually refers to the intrinsic electrical response of the device, which is almost instantaneous; from the perspective of biological analysis, response time also includes the diffusion and transport of target molecules, as well as the binding kinetics between probes and target molecules. These processes are usually rate-limiting steps, significantly extending the overall detection time [76,77,93,94]. This also suggests that interfacial molecular-recognition processes remain one of the key factors affecting practical sensing performance. Another point worth noting is that most of the studies listed in Table 2 were evaluated mainly in buffer systems. When detecting in real samples, some articles describe it as detectable current changes and avoid the issue of specific detection performance. In papers that provide actual detection data, the detection capability in real samples is often much lower than that in ideal environments. Therefore, the very high sensitivity reported under simplified conditions may not always be directly applicable to practical applications, where matrix effects and nonspecific interactions become more important.

## 4. Challenges in Practical Biosensing Applications

Although FET biosensors based on two-dimensional materials are often described as having high sensitivity and selectivity, these results are mostly obtained in buffer solution systems, and their performance often significantly decreases when applied to actual sample analysis. This indicates that the actual detection capability of two-dimensional material FET biosensors is significantly limited in complex sample environments. High ionic strength can cause charge screening effects, while nonspecific adsorption and interactions between biomolecules can introduce additional interference, jointly affecting the device response and reducing sensor sensitivity and stability [67,71]. In addition, current studies on 2D-material FET biosensors still focus heavily on relatively small recognition elements, such as aptamers and short nucleic acid probes. In practical protein sensing systems, however, antibodies and their target molecules are usually much larger and structurally more complex, and their binding events often already occur beyond the Debye length under physiological conditions. As a result, interfacial charge regulation alone is often insufficient for reliable electrical readout. This also explains why Debye-screening mitigation and signal-amplification strategies remain important topics in this section.

In practice, these effects are often reflected in signal attenuation, unstable response, increased noise, and reduced reproducibility [95]. A representative example is the performance of graphene-based FET biosensors in CRP detection. In complex systems such as artificial saliva, significant nonspecific effects were observed due to the complex composition of the sample. In this case, in order to obtain reliable detection results, preprocessing steps, including dilution and filtration, are usually required to reduce background interference, which contradicts the original intention of label-free rapid detection. Previous studies have shown that nonspecific interactions in complex matrices can significantly affect sensor response and increase interference. This example further illustrates that the excellent sensing performance obtained in buffer solution is difficult to fully maintain even in artificial simulation systems, and it is even more difficult to directly apply to actual biological samples. In these systems, matrix effects significantly limit detection capability [96]. From a mechanistic perspective, the core of these limitations lies in the signal transduction process at the solid–liquid interface, that is, how biological molecule recognition is converted into electrical signals. Due to the fact that this interface determines the modulation of channel charge distribution by molecular binding events, it directly affects the sensitivity, selectivity, and stability of FET biosensors [25]. In many cases, these problems are not caused by a single factor; rather, they are the result of the combined effects of multiple factors, such as charge screening, interface contamination, and environmental instability during the measurement process. From a practical application perspective, these issues can be summarized into three main aspects: Debye screening, nonspecific adsorption, and device signal drift and long-term stability issues. The following text will discuss these key issues and analyze them in conjunction with actual biological detection scenarios.

### 4.1. Debye Screening Effect in Complex Biological Media

Many of the studies discussed above either lack real-sample validation or show substantially weaker sensing signals in complex media than in buffer systems [97]. During the functionalization stage, it was previously mentioned that the recognition layer should not be excessively long and should also consider Debye-length limitations. The Debye length defines the effective range of electrostatic interactions that can be perceived and decreases with increasing ionic strength. Under physiological conditions, due to high ionic strength, the Debye length is usually less than 1 nm, and most probe lengths exceed this distance. When the operating distance exceeds this range, the interface potential modulation becomes strongly screened, making it difficult to effectively regulate the channel charge distribution, ultimately resulting in a significant decrease or even undetectable ΔVth modulation amplitude. Therefore, this remains one of the main obstacles limiting the practical application of two-dimensional-material FET biosensors [67].

In contrast, the size of most biomolecules, such as proteins or probe–target complexes, typically ranges from 2 to 10 nm [67,98]. Therefore, the electrical signals generated by target binding events usually occur outside the Debye length and are effectively screened, resulting in weak or undetectable signals. In practical testing, ionic strength is usually reduced by diluting the sample. However, due to the presence of many analytes at low concentrations, dilution further reduces signal strength, resulting in a trade-off between signal magnitude and screening reduction. Therefore, dilution alone cannot fundamentally solve this limitation. In addition, differences in probe design can affect the effective sensing distance [99]. For example, compared to DNA probes, peptide nucleic acid (PNA) probes have a neutral backbone and shorter effective recognition distance, which reduces background charge interference and allows binding events to occur closer to the sensing surface. Some studies compared the performance of DNA (0.1 × PBS) and PNA (1 × PBS) as probes under other identical conditions by monitoring the Dirac-point shift. Despite the high salt concentration in the PNA system, the LOD of the DNA probe was about 20 aM, while the LOD of the PNA probe reached 600 zM. This difference is consistent with the neutral backbone and shorter effective recognition distance of PNA, which favor binding events closer to the sensing surface. These results suggest that improving probe structure and reducing effective sensing distance can still enhance sensing performance even under relatively high ionic-strength conditions [73]. Therefore, compared with conventional DNA probes, PNA probes can reduce background charge interference and position target binding events closer to the sensing interface under high-ionic-strength conditions, thereby improving interfacial charge transduction and reducing the influence of Debye screening. One possible strategy for alleviating Debye screening is to place the probe molecule directly within the conductive pathway. In this design, a nanogap is introduced into the channel so that the probe can bridge the two electrodes. Before target binding, charge transport across the gap is weak. After binding, the probe may undergo a conformational change that modulates charge transfer through the gap, which can be detected as a change in current or resistance. Related mechanisms have been explored experimentally. For example, in nanogap-based structures, DNA hybridization can form conductive pathways between electrodes and thereby produce measurable conductivity changes [100]. This strategy, however, also has important limitations. First, the signal is highly sensitive to nanogap size and molecular configuration, which places strict demands on nanoscale fabrication precision. Secondly, the conductivity of biomolecules is often uncertain and strongly dependent on the local environment, making signal interpretation more difficult. Therefore, although nanoscale gap conduction can partially bypass Debye screening, it also weakens or even loses the relationship between molecular binding and ΔVth, and poses additional challenges in signal discrimination, as the measured signals may come from target binding, direct charge transport, or nonspecific interactions. Overall, overcoming Debye screening remains a fundamental challenge that requires a combination of probe design, interface engineering, and device structure optimization.

### 4.2. Nonspecific Adsorption and Surface Fouling

In real-sample detection, nonspecific adsorption is one of the main factors affecting sensor performance. Actual samples usually contain many biological components, including proteins, DNA, and RNA. These components, unrelated to the target analyte, may adsorb on the sensor surface, causing changes in current, increasing noise, and even false-positives and false-negatives, affecting the accuracy and reliability of detection [101,102].

In the process of functionalization, in addition to rationally designing the probe to enhance the selectivity for specific molecules, it is also necessary to reasonably optimize the density of the probe. An excessively high probe density may lead to overcrowding in the spatial structure of the probe and increase the possibility of nonspecific binding. An appropriate probe density will improve the specific binding efficiency and reduce the possibility of nonspecific binding [103]. At the device level, nonspecific adsorption can be reduced by introducing isolation or passivation layers. It can be mainly considered from the following aspects: The first is to introduce antifouling materials around the recognition layer to form an inert molecular coating. Antifouling materials such as PEG can effectively inhibit the nonspecific adsorption of proteins and other macromolecules, thereby improving the sensing performance [104]. The experimental studies have shown that PEG-modified surfaces can significantly reduce the nonspecific binding level of biomolecules and improve the selectivity and stability of the sensor [105]. The second is to isolate the detection area and non-detection area by physical or chemical methods. Using polydimethylsiloxane (PDMS) to build a microfluidic liquid cell, or using a substrate passivation layer, such as Al_2_O_3_, SiO_2_, or self-assembled monolayer (SAM) to shield the non-detection area, can effectively limit the area exposed to the electrolyte and reduce the interfacial stray adsorption [106,107]. This kind of structural optimization not only reduces the background current fluctuation, but also improves the repeatability and long-term stability [108].

### 4.3. Operational Stability and Signal Drift in 2D FET Biosensors

In the process of practical application, the stability of the sensor is one of the important factors that must be considered. Stability affects not only the accuracy and reproducibility of detection results, but also the suitability of the sensors for long-term monitoring, continuous detection, and operation in complex biological samples. This is particularly important in two-dimensional-material FET biosensors, where the channel material is generally exposed to the interfacial environment, and its electrical characteristics are highly sensitive to external conditions and potentially cause baseline drift, signal attenuation, or noise increase, thus affecting the accuracy of detection [22]. Firstly, the surface structure of two-dimensional materials may be affected by oxidation and degradation in air or water environments, resulting in threshold voltage drift and mobility decline [9]. To solve this problem, we can reduce the impact of environmental exposure by establishing isolation layers, optimizing substrate interfaces, or using heterostructures [109]. Secondly, in the electrolyte gate structure, the electrical double layer (EDL) and ion distribution may gradually become unstable over time, which will cause chronic baseline drift. Maintaining constant buffer and electrolyte conditions, including stable ionic strength and pH, together with optimization of the liquid–cell structure, can reduce instability caused by ion dynamics [110]. Finally, the instability of the functionalized layer is an important reason for the signal attenuation, including probe desorption, conformational changes, or biomolecular inactivation, which remains a significant and difficult problem to address [111]. Compared with physical adsorption, more covalent immobilization, multipoint connection, or the introduction of a spacer molecular layer can significantly improve the interface stability, and reasonable control of probe density can also help maintain the long-term response consistency. But at the same time, these methods, such as covalent modification, may change the carrier mobility and thus reduce the sensitivity [8,51].

In addition to the above key factors, there are also some relatively minor but non-negligible influencing factors, such as temperature fluctuation, electrode contact resistance change, and external electromagnetic interference [102,112]. These factors usually do not directly change the recognition mechanism, but will increase the noise level or cause short-term drift. It should be noted that these approaches, such as temperature control, packaging optimization, and electromagnetic shielding, are general engineering strategies commonly applied across various sensing platforms, rather than being specific to 2D FET biosensors. The overall signal stability can be further improved by engineering means such as thermostatic control, optimization of electrode packaging, use of differential measurement structure, and electromagnetic shielding [113].

**Figure 3:**
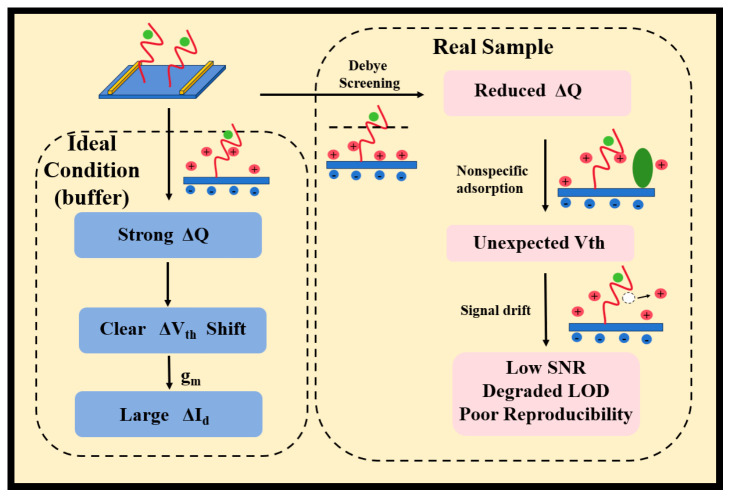
Disruption of the interface–device–performance pathway in 2D-material FET biosensors under complex biological environments.

Overall, the three key issues mentioned above—Debye screening, nonspecific adsorption, and signal drift or poor operational stability—essentially stem from interface charge shielding, environmental interference, and device instability (Figure 3). Rather than acting independently, these factors often influence different stages of the signal-transduction process simultaneously. Specifically, Debye screening reduces the effective interfacial charge modulation, nonspecific adsorption induces unintended potential variations, and signal drift further degrades the stability of the electrical output. These factors collectively weaken the effective signal transduction ability and lead to a decrease in signal-to-noise ratio (SNR), thereby limiting the detection reliability and practical sensing ability of FET biosensors in complex biological environments [25]. Therefore, although most of the above methods belong to general strategies that can improve overall signal stability to a certain extent, they have not fundamentally solved the inherent limitations of FET biosensors. In contrast, device level or interface design strategies such as electric double-layer (EDL) regulation are more in line with the inherent working mechanism of FET systems and are expected to provide more effective solutions.

## 5. Interface–Device Coupling Strategies for Signal-Transduction Enhancement

This section focuses on interface–device coupling strategies for overcoming Debye screening and enhancing signal amplification, thereby enabling more reliable biosensing in practical applications.

### 5.1. Interfacial Charge Regulation in Electrolyte-Gated FET Biosensors

The interface charge regulation in electrolyte-gated FET biosensors mainly targets the fundamental limitations caused by Debye shielding in high-ionic-strength environments. Under physiological conditions, the Debye length is usually less than 1 nm, much smaller than the size of most biomolecule complexes, resulting in effective shielding of the charge signal generated by target binding [17,71]. This makes it difficult for the corresponding biological signals to be effectively transduced into detectable signals even if molecular recognition processes occur. Therefore, the key issue of the interface layer is not only the functionalization of probes or molecular recognition itself, but also whether the interface charge disturbance can be effectively generated, retained, and fall within the detectable range [98].

**Figure 4:**
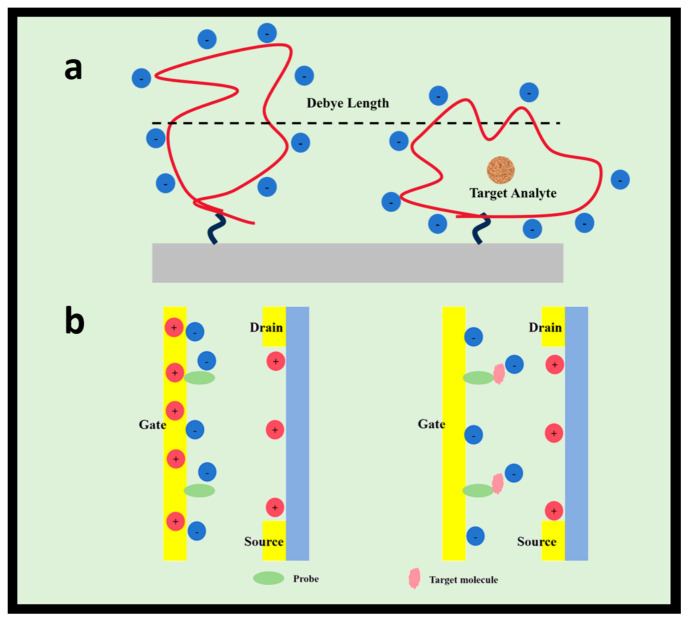
Electrostatic effects in electrolyte-gated FET biosensors. (**a**) Debye screening and binding-induced interfacial charge redistribution in an aptamer-functionalized FET. Adapted from ref. [114]. (**b**) Electric double layer (EDL) formation and signal-transduction mechanism in an electrolyte-gated FET. Adapted from Ref. [98].

Nakatsuka et al. functionalized ultrathin metal-oxide FET arrays with conformationally adaptive oligonucleotide stem-loop aptamers [114]. This technology is also of great reference significance for two-dimensional-material FETs. In this design, the aptamer receptor is fixed near the surface of the semiconductor channel. When the target molecule binds, the negatively charged phosphodiester backbone of the aptamer undergoes target-induced conformational rearrangement, and a large part of its charge density enters the Debye length, as shown in Figure 4a. Even in a high-salt buffer system, this mechanism can still produce a detectable electrical response.

In addition, when the nanoscale channels in the nanopore structure make contact with the solution, an EDL will be formed on the pore wall. Nanopore-based strategies mainly rely on ion-transport dynamics and overlapping EDL effects within nanoscale channels to modulate ionic-current signals [115]. When studying the electrical properties of electrically controlled nanopores, Liu et al. found that in nanopores with a diameter much larger than the Debye length, an obvious phenomenon of electric-field modulating current can still be observed, and this modulation far exceeded the Debye-length limit [116]. Therefore, although the nanopore method cannot completely eliminate the Debye shielding itself, it can make the electric-field effect far exceed the Debye-length limit in the nanoscale channel through the descreening effect caused by strong ion transport and modulate the signal by changing the potential and current in the nanopore. This phenomenon provides useful insight into overcoming Debye-screening limitations during biosensing. Although the descreening effect in nanopore systems does not originate from intrinsic interfacial charge modulation, it can still alter the effective electrostatic potential at the channel interface. As a result, the induced changes can be quantitatively reflected by shifts in threshold voltage (ΔVth), making Vth a useful parameter to evaluate the effectiveness of descreening strategies.

At present, Debye shielding is the core limitation of two-dimensional-material FET in actual detection, especially in physiological environments such as blood. Its essence comes from the electrostatic shielding effect of ions in electrolytes on target charges. Overall, in practical applications, the strategies to alleviate Debye shielding can be considered from two aspects: shortening the effective charge action distance and improving the charge resolution of devices. With the continuous development of more advanced materials and the development of channel structure toward more complex and efficient designs, the influence of Debye shielding effect is expected to gradually decrease. Meanwhile, as an important component of interface regulation, the functionalization strategy is closely related to device structure. Introducing adjustable conformational units such as hairpin structures during functionalization is also a feasible method to reduce Debye influence [114].

### 5.2. Device-Level Signal Amplification in FET Biosensors

As mentioned above, interface charge regulation mainly determines whether the charge disturbance caused by target binding can be effectively generated and enter the detectable range under Debye-shielding conditions [71]. However, even when these interfacial signals are preserved, their intensity is often still too weak for stable electrical readout. Therefore, beyond interface regulation, device-level engineering strategies are also important for improving signal amplification and readout capability [22]. In this process, weak interfacial charge perturbations can be converted into measurable electrical outputs through improved transconductance, optimized carrier transport, and enhanced signal-to-noise ratio [114]. Controlled doping and defect engineering can regulate the electrical properties of 2D materials by introducing heteroatoms, vacancies, or local defect sites. These modifications may influence carrier transport, threshold voltage, and transconductance, further affecting signal amplification and electrical response in FET biosensors. In addition, engineering the surface structure and morphology of materials is also an effective strategy for improving interfacial signal transduction [63,64,65]. Nik Zulkarnine et al. developed an on-chip nano-corrugated graphene (NCGr)-based multimodal biosensing platform and observed that the electric double-layer (EDL) capacitance decreased from approximately 2.94 μF cm^−2^ to 2.43 μF cm^−2^ as the corrugation depth increased, while the charge-transfer resistance also increased [117]. These results suggest that nanoscale surface morphology can influence interfacial charge transfer and local ionic screening behavior, enabling coupled electrochemical and field-effect signal transduction. This morphology-engineering strategy may also be applicable to 2D-material FET biosensors in the future for improving signal transduction under Debye-screening conditions.

Chu et al. used the electric double layer (EDL) as an electrolyte gating mechanism to improve the charge coupling of the interface, so that the influence of interfacial binding on the channel is strongly modulated by the EDL and solution capacitance [98]. They used ultrashort pulse measurement, so that the change in interface potential can be rapidly transmitted to the channel, thereby partially reducing the influence of Debye screening, as shown in Figure 4b. This method can also obtain stable electrical signals at higher ionic strengths. Under the applied grid bias voltage, counter ions accumulate near the grid surface, and ions with opposite symbols accumulate near the channel, forming a tightly coupled EDL, generating a strong local electric field. When the target molecule is combined with the probe at the interface, this disturbance of the interface charge is rapidly coupled to the channel through EDL. Even in the high-ionic-strength environment, where traditional Debye screening will inhibit long-range electrostatic interactions, the generated electrical signal can be transmitted more effectively and rapidly. Although the focus of this article is not on the detection performance, this method realizes the effective detection of NT-proBNP and CRP in undiluted human serum, requiring only a 5 min response time, which has important reference significance in improving the performance of two-dimensional-material FET [98,118].

**Figure 5:**
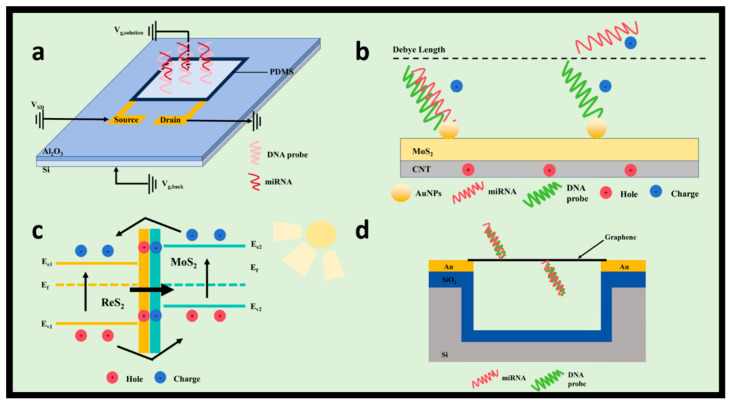
Representative device-engineering strategies in 2D-material FET biosensors. (**a**) Dual-gate GO/graphene solution-gated transistor (SGGT) structure. Adapted from ref. [123]. (**b**) SWCNT/MoS_2_ heterostructure FET with AuNP-assisted interfacial coupling structure. Adapted from Ref. [125]. (**c**) Optically assisted ReS_2_–MoS_2_ heterojunction FET structure. Adapted from Ref. [78]. (**d**) Suspended-graphene FET structure. Adapted from Ref. [133].

The double-gate field-effect transistor is a new structure design which can regulate the channel by introducing two gates. Compared with the traditional single-gate structure, the double-gate structure can significantly enhance the control ability of channel potential through the coupling regulation of the top solution gate and the bottom back gate [119]. The introduction of back gate bias can optimize the operating point of the channel and improve the transconductance of the device, thus enhancing the sensitivity of channel carriers to interface charge perturbation. From the electrostatic point of view, the double-gate structure effectively increases the gate capacitance coupling, thereby amplifying the interface potential disturbance and improving the efficiency of electrostatic-to-electrical-signal conversion. Therefore, when the target molecules combine to cause the redistribution of interfacial charges, the weak potential changes generated by them can be more efficiently converted into detectable electrical signals to achieve the electrical-amplification effect [120,121]. Kammarchedu et al. constructed a dual-gate graphene field-effect transistor, which achieves capacitive amplification of interface signals by coupling a high-k dielectric back gate with an electrolyte top gate and introducing feedback regulation [122]. This device exhibits significant signal-enhancement capability (up to about 20 times), while increasing signal-to-noise ratio by about seven times and achieving over 15 times the signal-drift suppression. From a device perspective, this performance improvement can be attributed to the enhanced transconductance and gate coupling ability under dual-gate modulation, which enables interface charge disturbances to be more efficiently converted into electrical signals. The synergistic improvement of signal amplification and noise suppression indicates that this structure has significant advantages in optimizing sensing performance. In miRNA-21 detection, Chi Hsien Huang constructed a GO/G bilayer solution gate graphene transistor (SGGT). Among the layers, the upper GO is rich in oxygen-containing functional groups such as carboxyl groups, which can stabilize the amino-modified DNA probe through covalent bonds to achieve high-density and stable probe functionalization. The lower graphene maintains a complete sp^2^-conjugated structure and excellent carrier mobility as an efficient charge-transport channel; the structure is shown in Figure 5a. PDMS constructs a micro-liquid pool on the device’s surface to limit the electrolyte area and prevent leakage. The amino-modified DNA probe is covalently immobilized on the surface of GO, and the lower graphene acts as a conductive channel to realize the transmission and amplification of electrical signals. Three voltages were simultaneously applied to the device. From top to bottom, the solution-gate voltage (Vg, solution) directly acts on the droplet through the reference electrode in the electrolyte, the source-drain voltage (VSD) is used to drive the channel current, and the back-gate voltage (Vg, back) is coupled through the Al_2_O_3_ insulating layer to regulate the channel-carrier concentration, so as to optimize the device-operating point and enhance the signal response [123]. When only a solution gate is applied, the sensitivity is 19.26 mV/dec. After applying a back-gate bias of −0.1 V, the sensitivity increased to 33.65 mV/dec, an increase of over 70%, while maintaining excellent linear response. This performance improvement reflects the enhancement of transconductance and capacitance coupling ability under dual-gate modulation, thereby more effectively converting interface charge disturbances into electrical signals. On this basis, the device achieved a low detection limit of 10 fM, indicating that transconductance enhancement plays an important role in improving sensing performance [123].

Two-dimensional-material heterostructures have received widespread attention in FET biosensors. By stacking different two-dimensional materials in layers, specific band structures and built-in electric fields can be formed at the interface, significantly regulating the transport behavior of charge carriers [124]. Compared with a single material, heterostructures can simultaneously combine high-mobility materials with semiconducting materials with finite band gaps, enhancing their responsiveness to interface-charge changes while maintaining excellent conductivity properties [20].

Youqiang Xing used SWCNTs as the channel material for FETs, grew MoS_2_ thin films on the surface of SWCNTs by precisely controlled atomic layer deposition (ALD), and constructed an SWCNT&MoS_2_ heterostructure [125]. The strong charge coupling between SWCNT and MoS_2_ can be achieved by adjusting the number of ALD cycles (Figure 5b). The upper MoS_2_ serves as a functionalized interface for the immobilization of DNA probes, and the lower CNT network serves as a high-mobility charge-transport channel. AuNPs act as a molecular bridge and spacer between the probe and the channel, effectively bringing the sensing interface closer to the Debye-length range, thereby enhancing the transduction efficiency of interfacial charge perturbation. When the negatively charged miRNA hybridizes with the DNA probe within the Debye-length range, it will cause the redistribution of interfacial charges and upward bending of the energy band, resulting in the accumulation of holes in the p-type CNT channel, which is manifested by the increase in device current. When the charge change occurs outside the Debye length, its potential disturbance will be significantly shielded by the electrolyte solution, which is difficult to effectively convert into detectable electrical signals. Under different preparation conditions, the heterostructure device shows the best electrical performance, including higher field-effect mobility and better on/off current ratio, when the number of ALD cycles is three. In the process of biosensing, when the interface charge disturbance caused by the adsorption of target molecules on the device surface exceeds the Debye shielding length of the sensor, the potential change caused by it will be difficult effectively detect [98]. To partially overcome this limitation, AuNPs were used as a bridge between the probe and the channel film, effectively expanding the Debye-shielding range of the device and enhancing the interfacial charge transduction [126]. When negatively charged biomolecules adsorb at the sensing interface, it will cause the energy band to bend upward, which leads to the accumulation of holes in the single-walled carbon nanotube channel. Therefore, the enhanced response of device current was observed in SWCNT FETs decorated with AuNPs. Based on this heterostructure design, the device achieved a linear detection range of 1 × 10^−17^ to 1 × 10^−10^ mol·L^−1^ for miRNA-21 and exhibited ultra-high sensitivity, with a theoretical minimum detection limit of 1.9 × 10^−18^ mol·L^−1^ [126]. Overall, compared to traditional FET devices, heterojunction FET biosensors exhibit significantly enhanced signal-transduction capabilities, with performance improvements typically reflected in several-orders-of-magnitude increases in current sensitivity, mainly due to the enhanced carrier modulation ability introduced by heterojunctions [127,128].

The rapid development of two-dimensional materials in the field of optoelectronic devices in recent years shows that the optical-assisted strategy is expected to further improve the sensing performance. Two-dimensional materials can regulate the channel carrier concentration through photogating effects, photoconductive gain, or light-induced charge transfer under light conditions, and amplify the surface potential disturbance caused by biomolecule adsorption, so as to improve the signal-to-noise ratio and detection sensitivity [129,130,131]. Xing et al. formed a superlattice structure on the basis of the ReS_2_–MoS_2_ heterojunction nanotube structure. The two materials were alternately arranged in a periodic and regular manner to form a special heterostructure [78]. Compared with the formation of a single type-II-band-aligned double-layer heterostructure, the ReS_2_–MoS_2_–ReS_2_ three-layer structure can build two symmetrical type-II interfaces, thereby significantly enhancing the efficiency of charge separation and interlayer exciton generation and improving the density and transport capability of carriers involved in transport. The built-in electric field at the heterointerface induces band bending and carrier redistribution, forming a spatially separated charge-transport pathway that enhances sensitivity. A schematic diagram of the energy-band structure and carrier separation of the ReS_2_–MoS_2_ heterostructure interface under light irradiation is shown in Figure 5c. Due to the carrier concentration gradient between the two materials, a depletion zone and a built-in electric field are formed at the interface, and the direction of the electric field is from ReS_2_ to MoS_2_. After the electron–hole pairs are generated by light excitation, the electrons are transferred from the conduction band of MoS_2_ to ReS_2_, and the holes are transferred from the valence band of ReS_2_ to MoS_2_ under the action of the built-in electric field, thus promoting charge separation and improving the carrier migration ability [132]. Ef denotes the Fermi level, and EV and EC denote valence band and conduction band levels, respectively. Detection was achieved by depositing AuNPs onto the surface of the heterostructure and then fixing the probe by Au-S On the basis of these. This strategy partially alleviated the impact of Debye screening by increasing the sensitivity of the device to charge with an external light source. The linear range is 10 aM to 1 nM, the LOD is 2.1 aM, and the RSD of the experiment is 1.5% [78].

The suspended structure began to be used for gas adsorption to reduce noise, increase surface exposure area, and improve charge-transport characteristics [133,134]. It plays an important role in the detection of biomolecules by selecting appropriate functionalization methods. Zhiming Deng et al. fabricated a suspended-graphene FET structure [133]. They formed a cavity under graphene by etching SiO_2_, thereby suspending the graphene channel. The schematic structure of the suspended-graphene FET for miRNA-21 detection is shown in Figure 5d. By etching the SiO_2_ layer to form a cavity on the Si substrate, the graphene channel is suspended, thereby reducing the substrate-charge scattering. Au electrodes serve as the source and drain contacts. The DNA probe immobilized on the surface of graphene hybridizes specifically with the target miRNA-21, generates surface-charge perturbation, and modulates the carrier concentration of graphene channel to achieve detection. They realized functionalization by π–π stacking of PBASE and graphene, and then detected it by covalent reaction of its NHS ester group with amino-modified DNA probe. The concentration of standard miR-21 ranged from 5 fM to 50 pM. The LOD of the suspended device was determined to be 7 fM, which was ten times lower than the 76 fM LOD of the traditional device.

As discussed above, the key role of device-layer engineering strategies in signal amplification and electrical signal readout was discussed [22]. By combining dual-gate regulation, heterojunction design, and optoelectronic coupling device structures, effective amplification and efficient signal transduction of weak interface disturbances can be achieved [129].

Interface charge regulation governs whether the charge disturbance induced by target molecule binding can be effectively generated, maintained, and fall within the detectable range under Debye-screening conditions [71]. In parallel, device-level engineering dictates how these interfacial perturbations are transduced and amplified into measurable electrical signals with sufficient signal-to-noise ratio [116]. Although these aspects are discussed separately for structural clarity, the overall sensing response can be approximated within a simplified electrical framework as a cascaded yet interdependent process involving interfacial charge generation and device-level signal amplification [18,53].

To systematically illustrate how different strategies influence signal transduction in 2D FET biosensors, representative interfacial and device-engineering approaches are summarized in Table 3. These strategies mainly regulate the interface charge disturbance (ΔQ) and device amplification capability (gm), which together influence the overall signal transduction behavior. In this context, the interfacial response may be approximately expressed as ΔVth = ΔQ/Ceff, while the device amplification capability is characterized by the transconductance gm, leading to an output current variation of ΔId ≈ gm · ΔVth. These relationships are mainly intended as simplified conceptual descriptions rather than rigorous quantitative models. It should be noted that, in practical systems, parameters such as Ceff and gm are not strictly independent of ΔQ, as interfacial electrostatics and operating bias conditions can dynamically influence these quantities. Consequently, the sensing performance is jointly governed by the magnitude of interfacial charge perturbation (ΔQ) and the effective transduction capability of the device (gm), which are inherently coupled through the device physics. Hwang et al. demonstrated that deformed graphene structures can induce a substantial shift in the Dirac point (ΔVDirac ≈ 40 mV), enabling biosensing beyond the Debye length through enhanced interfacial charge coupling [72]. In graphene-based FET biosensors, ΔVDirac is often used as an analogous indicator of threshold-voltage variation (ΔVth) in conventional semiconducting channels. Although this strategy is realized via device-level structural modification, its main effect lies in strengthening interfacial charge coupling and partially alleviating electrostatic screening, thereby increasing the effective input signal (ΔVth) and improving overall transduction efficiency. This example further suggests that interfacial processes and device-level regulation often influence sensing behavior in a coupled manner rather than as completely independent effects.

## 6. Conclusions

FET biosensors based on two-dimensional materials exhibit strong potential in high-sensitivity and label-free detection due to their excellent electrical performance and inherent signal-amplification ability. They demonstrate strong detection performance in ideal environments for nucleic acid, protein, and small-molecule detection. However, in the actual detection process, complex preprocessing steps such as dilution and purification are still required to obtain reliable sensing results. This is mainly limited by factors such as Debye screening, nonspecific adsorption, stability, and signal drift [67,101]. These challenges indicate that the development of 2D-material FET biosensors in the future should not only focus on improving detection performance such as detection limits, but also rely on the synergistic optimization of interface chemistry and device structure, including conformationally responsive probes; stronger charge coupling capabilities; and advanced designs, such as dual-gate structures, heterojunctions, and optoelectronic-assisted configurations, to improve practical sample detection capability and reduce dependence on extensive sample preprocessing.

Linking surface functionalization, electronic structure modulation, and device response is crucial for understanding sensing mechanisms, but establishing a unified quantitative or predictive model is quite difficult due to the complexity of electrolyte environments, molecular interactions, and sample environments. In this review, we discuss a simplified framework for describing the relationship between interfacial processes, device response, and sensing performance. We select representative electrical parameters, where Vth represents interface effects, gm reflects device amplification capability, and Id represents final sensing output performance. This framework is mainly intended as a conceptual description of how interfacial regulation influences device behavior and sensing response, rather than a rigorous predictive model or parameter-extraction method. At present, many studies have focused on the changes in Vth caused by the binding of target molecules, but this is mainly used to demonstrate the occurrence of binding and conformational changes, and less attention has been paid to the impact of functionalization itself on Vth, resulting in a lack of unified understanding. It is expected that these aspects will be more widely applied in future research to facilitate comparisons between different works. From an interdisciplinary perspective, the interface–device–performance framework provides a useful basis for connecting interface chemistry, device physics, and analytical performance, and offers a common perspective for understanding different sensing systems. Meanwhile, it should be pointed out that current reporting practices for key device characteristics and performance metrics remain inconsistent, thus making direct comparison across different studies difficult. For example, different studies often use inconsistent criteria to define and evaluate device-response and stability-related parameters, thus making direct comparison difficult and hindering the establishment of a unified evaluation framework. Therefore, for practical applications, more standardized characterization and reporting of device behavior and sensing performance are needed. In addition to LOD, future studies should also report key sensing parameters, such as linear range, real-sample recovery, relative standard deviation (RSD), operational stability, and overall detection time, whenever possible. It would also be helpful to include the influence of functionalization on device electrical behavior, for example, threshold-voltage (Vth) shift, carrier-transport characteristics, or transconductance changes, to better understand the relationship between interface regulation and sensing response. More consistent reporting of these parameters may improve comparison between different studies and provide a clearer basis for evaluating the practical applicability of 2D-material FET biosensors.

## Figures and Tables

**Table 1:** Comparative analysis of functionalization strategies in 2D-material FET biosensors.

Functionalization Strategy	Primary Mechanism	Interfacial Charge Behavior	Band/Fermi Level Effect	Channel Modulation Pathway	Key Feature	References
Radical grafting	Covalent sp^3^ defect formation	Charge transfer at bonding sites	Localized states, DOS modification	Defects → scattering → conductance change	Structural modification, high stability	[29,30,31,32]
Cycloaddition	π-bond covalent reaction	Charge redistribution in bonding region	Local band distortion	Covalent bond → electronic perturbation	Partial π-conjugation preserved	[34,35,36,37]
Michael/nucleophilic addition	Defect-site reaction	Localized charge transfer	Formation of localized electronic states	Defect sites → local conductance change	Site-selective functionalization	[39,40,41,42]
Silanization	Surface bonding + dipole layer	Dipole-induced potential shift	Interfacial band bending	Dipole → potential → carrier modulation	Stable surface anchoring	[48,49,50,51]
Polymer grafting	Polymer-coating layer	Charge screening/distribution	Minimal band impact	Distance increase → field attenuation	Debye-screening limitation	[52,53,54]
Π–π stacking	π–π orbital interaction	Interfacial charge transfer	Fermi-level shift (no lattice damage)	Surface adsorption → charge modulation → current change	Preserves intrinsic structure	[60,61,62]
Electrostatic adsorption	Coulomb interaction	Surface charge-density change	Fermi-level modulation	Charge → electric field → carrier modulation	Fast and reversible	[8,11,35]

**Table 2:** Representative performance of 2D material-based FET biosensors for biomolecular detection.

Category	Target	Channel Material	LOD	Linear Range	Response Time	Reference
Nucleic acids	DNA	Deformed Graphene	~0.6 aM	—	1 h	[73]
	miRNA-21	Graphene- MoS_2_	6.06 fM	10 fM–10 nM	~30 min	[76]
	miRNA-155		2.59 fM			
	miRNA-208a	Graphene	5.3 fM	—	~40 min	[77]
	miRNA-21	ReS_2_–MoS_2_	2.1 aM	10 aM–1 nM	—	[78]
	miRNA-21	rGO	4.38 fM	10 fM–1 nM	—	[79]
	miRNA-486-5p		6.4 fM			
	miRNA-155		2.47 fM			
	miRNA-205		2.36 fM			
	miRNA-21	PtNWs@MXene	0.84 fM	—	—	[80]
Proteins	IFN-γ	MoS_2_	59.8 fM	2 pM–250 nM	—	[83]
	Tau	MoS_2_	* 0.2 fM	* 0.2 fM–20 pM	—	[85]
	Spike protein	WSe_2_	* 140 fM	* 140 fM–56 nM	real time	[86]
	Streptavidin	MoTe_2_	1 pM	1–10 pM	20 s	[87]
	HIgG antigen	Graphene–MoS_2_	* 0.083 fM		—	[20]
		MoS_2_–graphene	* 0.176 fM			
Small molecules	Cortisol	Black phosphorus	1 aM	100 aM–10 nM	tens of seconds	[88]
	Cortisol	Graphene	500 fM	500 fM–100 nM	20 s	[89]
	Dopamine	Graphene	60 zM	0.1 zM–10 pM	—	[90]
Ions	Hg^2+^	Graphene	20 pM	100 pM–100 nM	—	[91]

Protein concentrations are typically reported in mass-based units (e.g., ng mL^−1^), and the molar values marked with “*” are converted based on reported molecular weights for comparison. Only representative examples discussed in this review are listed here, rather than a complete summary of all reported devices.

**Table 3:** Representative interfacial and device engineering strategies in 2D FET biosensors and their roles in signal transduction.

Engineering Category	Strategy	Affected Parameter	Mechanism	Reference
Interfacial engineering	Covalent functionalization (e.g., silanization, diazonium)	ΔQ	Strong charge transfer, stable immobilization	[28,29,30,31,32,33,48,49,50,51]
	Noncovalent functionalization (e.g., PBASE and π–π stacking)	ΔQ	Charge redistribution without lattice damage	[60,61,62]
	Probe design (PNA and short probes)	ΔQ	Reduced sensing distance, enhanced effective charge	[73,99]
	AuNP-assisted coupling	ΔQ to ΔVth	Charge bridging and signal amplification	[125,126]
	PEG/antifouling layer	ΔId (SNR)	Suppression of nonspecific adsorption and noise	[105,106]
	EDL regulation	ΔVth	Enhanced interfacial capacitance coupling	[98,119]
	Nanogap/nanopore structures	ΔQ/ΔVth	Descreening effect, direct charge transport	[101,118]
	Aptamer conformational switching	ΔVth	Structural change induces potential variation	[115]
Device engineering	Dual-gate structure	gm	Enhanced gate coupling and transconductance	[120,121]
	Heterostructures (e.g., MoS_2_/graphene)	gm	Improved carrier transport and band alignment	[20,125,127,128,129]
	Optoelectronic coupling	gm/ΔId	Photo-induced carrier modulation	[78,130,131]
	Suspended channel structure	ΔId (SNR)	Reduced substrate scattering and noise	[133,134]
	Microfluidic integration	ΔId/stability	Controlled sample environment	[78,107,108,109]
	Passivation layers (e.g., Al_2_O_3_)	ΔId	Noise suppression and improved stability	[107,108,109]
Others	AI/signal processing	ΔId (SNR)	Signal extraction and noise filtering	[115]

## Data Availability

Not applicable.
